# Study on the Mechanisms of Flavor Compound Changes During the Lactic Fermentation Process of Peach and Apricot Mixed Juice

**DOI:** 10.3390/foods13233835

**Published:** 2024-11-28

**Authors:** Yao Zhao, Ruoqing Liu, Ying Mu, Mingshan Lv, Jun Xing, Li Zheng, Aihemaitijiang Aihaiti, Liang Wang

**Affiliations:** School of Life Science and Technology, Xinjiang University, Urumqi 830046, China; agneslll@163.com (Y.Z.); liuruoqing6688@163.com (R.L.); muying2208@126.com (Y.M.); lvmingshan6666@163.com (M.L.); lx1729580226@163.com (A.A.)

**Keywords:** apricot, flavor, flat peach, lactic acid bacteria, lactic fermentation, mixed juice

## Abstract

This study employed headspace solid-phase microextraction gas chromatography–mass spectrometry (HS-SPME-GC-MS) and liquid chromatography–mass spectrometry (LC-MS) for non-targeted metabolomics analyses to examine the impact of mixed fermentation with various lactic acid bacteria (LAB) on the flavor compounds and metabolites of peach and apricot mixed juice (PAMJ), specifically focusing on the alterations of volatile compounds and non-volatile metabolites, as well as their metabolic pathways during the fermentation process. A total of 185 volatiles were identified using HS-SPME-GC-MS analysis, revealing significant differential metabolites, including eugenol, benzaldehyde, and γ-decalactone etc. The results indicated that lactic fermentation significantly enhanced the overall flavor of the juice toward the end of the fermentation process. In the interim, untargeted metabolomics utilizing LC-MS identified 1846 divergent metabolites, with 564 exhibiting up-regulation and 1282 demonstrating down-regulation. The metabolic pathway study performed by the Kyoto Encyclopedia of Genes and Genomes (KEGG) revealed significant changes in the metabolic levels of amino acids and saccharides after the lactic fermentation of PAMJ. Primarily associated with amino acid metabolism and starch and sucrose metabolism pathways. This work establishes a theoretical foundation for advancing fermented fruit juices with superior quality.

## 1. Introduction

Flat peaches and small white apricots belong to the Rosaceae family and have a cultivation history in China that spans over two thousand years [1]. Flat peaches and white apricots from Xinjiang are highly esteemed for their succulent flavor and appealing hue. Flat peaches include elevated levels of vitamins A, B, and C, along with various trace elements such as iron, calcium, and phosphorus. In contrast, white apricots are abundant in vitamin C, β-carotene, and phenolics [2,3]. The two fruits provide significant economic and nutritional worth and are preferred by customers. Flat peaches and white apricots are characteristic seasonal fruits with delicate skin and meat, making them susceptible to mechanical injury and microbiological contamination post-harvest [4]. Extended refrigeration and cold-chain transportation adversely impact fruit flavor, freshness, and safety of fruit. These treatments may result in reduced flavor, the buildup of deleterious substances (including *Penicillium* and *Aspergillus* as well as bacteria such as *Salmonella* and *E. coli*), and a deterioration in overall quality, which can negatively impact consumer purchasing power and the exportation of fresh fruit [5]. Although methods such as pasteurization, heating, and adding preservatives are widely used to extend the shelf life of processed fruit products, these same techniques may trigger negative changes in physicochemical characteristics and flavor [6]. For example, added chemical preservatives (sulfates and benzoic acid) may interact with the natural components of the fruit and may form compounds that are potentially harmful to health. Furthermore, high-temperature processing can result in the breakdown of natural antioxidants (e.g., polyphenols), potentially diminishing the antioxidant activity of the fruit. Studies have shown that peaches may develop undesirable odors after heat treatment, which may stem from certain microorganisms remaining alive after heat treatment, leading to the development of off-flavors [7]. Furthermore, it was determined that the degradation of flavor in apricots during extended storage at low temperatures was primarily associated with alterations in aroma volatiles [8]. Consequently, mixed juice not only improves the problems with single-juice lactic fermentation but also provides a lot of nutrients, better quality, and strong flavor, demonstrating significant application potential [9]. Lactic fermentation of fruit juices maintains vital nutrients, degrades toxic compounds, and generates various new active substances, enhancing the flavor of the food. Lactic fermentation prolongs the shelf life of fruit and vegetable juices, suppresses the proliferation of unwanted microorganisms, and prevents flavor degradation caused by processing methods like autoclaving, hence improving the overall quality of fermented foods [10].

Fermentation is recognized as an effective technological process for producing various fruit-derived products, including alcoholic beverages, fruit vinegar, and fermented fruit juices. LABs are pivotal metabolite producers in this context, as they can introduce novel sensory properties and enhance the nutritional profile of food products. Comprehensive studies have shown that the fermentation of fruit juices by Lactobacillus species results in significant changes in the metabolite composition, encompassing organic acids, sugars, alcohols, esters, and various flavor compounds, which collectively improve the flavor profile of the fermented fruit juices [11]. The fermentation of tomato juice [12] by *L*. *plantarum* and *L*. *casei* led to significant increases in ketones, alcohols, and acids while concurrently reducing hydrocarbons, aldehydes, and esters levels. Furthermore, the fermentation of fruit and vegetable juices [13] by the heterofermentative strain *L*. *fermentum* increased acetic acid generation, enhancing the desirable sour flavor profile. Other strains, including *L*. *casei* and *L*. *rhamnosus*, were seen to augment the concentration of essential volatile components such as acetoin and diacetyl, noted for their buttery aroma and flavor [13].

Recent studies have indicated a burgeoning interest in elucidating the underlying mechanisms of flavor modification in fermented foods. For instance, peach juice fermented with *L*. *acidophilus* exhibited enriched nutritional and flavor attributes compared to its unfermented counterpart. Additionally, *L*. *plantarum* demonstrated superior biotransformation capabilities and flavor-enhancing properties [14]. Significant flavor and nutritional content improvements were also observed in jujube juice fermented with *Saccharomyces cerevisiae*, with analyses revealing that the metabolism of alanine and aspartic acid predominantly influenced early flavor alterations. In contrast, starch and sugar metabolism and glutathione metabolism pathways, emerged as significant contributors to flavor changes in the later stages of the fermentation process [11]. Despite these advancements, research investigating the fermentation of peaches and apricots by LAB remains comparatively sparse, with prior studies predominantly concentrating on flavor component alterations and insufficient emphasis on metabolite changes and potential mechanisms governing flavor development post-fermentation. Furthermore, there is a notable gap in the literature regarding the fermentation of mixed juices utilizing mixtures of various Lactobacillus species. Consequently, we hypothesize that distinct Lactobacillus species may significantly influence on flavor dynamics during the fermentation of mixed liquid matrices. To this end, we undertook metabolomic studies to elucidate the specific contributions of two fruit-mixed juices to mutual flavor enhancement.

In this experiment, five LAB strains were used to ferment PAMJ, and GC-MS observed the changes in flavor during lactic fermentation. Meanwhile, LC-MS was applied to explore the changes in metabolic profiles before and after lactic fermentation to search for critical metabolites, thus elucidating the flavor metabolism mechanism of the mixed fermented juice.

## 2. Materials and Methods

White apricots (local sales market in Hotan, Xinjiang, China), flat peaches (local sales market in Yili, Xinjiang, China), the same batch of materials was used in the experimental process. *L*. *acidophilus* CICC 6086, *L*. *brevis* CICC 20014, *L*. *fermentum* CICC 25124, *L*. *reuteri* CICC 6123, *L*. *plantarum* CICC 25125, *and L*. *paracasei subsp*. *Paracasei* CICC 6109 (China center of industrial culture collection, Beijing, China). All strains were activated twice and incubated at 37 °C for 24 h before use. Pectinase (500 u/g), cellulase (50 u/mg), and hemicellulose (20 u/mg) (yuanye Bio-Technology Co., Ltd., Shanghai, China).

### 2.1. Preparation and Fermentation of PAMJ

First, mature white apricots and peaches were selected, and deformed or diseased fruits were eliminated, washed manually, pitted, and homogenized at 25 °C. After homogenization, PAMJ were obtained by mixing at the ratio of peach pulp to apricot pulp = 2.5:1. To the sample, 0.2% (*w*/*v*) pectinase, 0.1% (*w*/*v*) cellulase, and 0.1% (*w*/*v*) hemicellulase were added, and the enzyme digestion was carried out at 55 °C for 3 h. The total soluble solids (TSS) were adjusted to 16 Brix, and then the sample was pasteurized at 90 °C for 30 min. After cooling, the strains, with an inoculum volume of 5.2 × 10^6^ CFU/mL, were introduced into the sterilized fruit pulp. The inoculation proportions of PAMJ were as follows: *L*. *fermentum* (15% *v*/*v*), *L*. *acidophilus* (1% *v*/*v*), *L*. *reuteri* (15% *v*/*v*), *L*. *paracasei subsp*. *paracasei* (13% *v*/*v*), *L*. *plantarum* (13% *v*/*v*), and *L*. *brevis* (34% *v*/*v*). They were exposed to a constant temperature oscillation incubator (ZD-85A, Changzhou, China) and regulated at 37 °C for 20 h of anaerobic lactic fermentation. At the end of fermentation, the filtrate was filtered and sterilized at 90 °C for 30 min and then canned.

#### Determination of Physical and Chemical Properties

Determination of PAMJ pH value: GB/T 10467-1989 [15]; PAMJ total acid content: refer to GB 12456-2021 [16] “Determination of Total Acid in Foods” methodology; determination of soluble solids content in PAMJ: GB/T 12143-2008 [17]; the experiment was repeated three times. The mean and standard deviation were calculated and recorded.

### 2.2. HS-SPME-GC-MS Analysis

We modified the method of Zhou et al. [18]. The extraction conditions of HS-SPME were as follows: at a constant temperature of 60 °C for 5 min with shaking; a 120 μm DVB/CWRIPDMS extraction head was inserted into the headspace vial of the sample, headspace extraction was conducted for 15 min, followed by sample resolution at 250 °C for 5 min, after which separation and identification were performed using GC-MS. The extractor head was subjected to aging in a fiber conditioning station at 250 °C for 5 min prior to sampling. Chromatographic parameters: DB-5MS capillary column (30 m × 0.25 mm × 0.25 µm, Agilent J&W Scientific, Folsom, CA, USA), high-purity helium carrier gas (purity ≥ 99.99%), constant flow rate of 1.2 m/min, inlet temperature of 250 °C, splitless injection, solvent delay of 3.5 min. The temperature was sustained at 40 °C for 3.5 min, subsequently elevated to 100 °C at a rate of 10 °C/min, then to 180 °C at 7 °C/min, and ultimately to 280 °C at 25 °C/min, where it was held for 5 min.

Mass spectrometry parameters: electron impact ionization source (EI), ion source temperature 230 °C, quadrupole temperature 150 °C, mass spectrometry interface temperature 280 °C, electron energy 70 eV, scanning mode in selected ion monitoring (SIM), qualitative and quantitative ion analysis with precise scanning (GB 23200.8-2016 [19]).

### 2.3. Analysis of Relative Odor Activity Values

The relative odor activity value (rOAV) was employed to assess the distinctive aromatic components of PAMJ during the lactic fermentation phase. A compound is classified as a characteristic aroma compound when its rOAV ≥ 1. rOAV represents the relative content/odor threshold value, with the threshold value derived from existing literature [20,21].

### 2.4. Non-Targeted Metabolomics

Sample pretreatment: 50 mL of sample from a 50 mL EP tube was mixed thoroughly with 600 μL of 70% (*v*/*v*) methanol containing internal standard extract as internal standard. The sample was vortexed for 30 s, repeated six times, and subsequently allowed to stand for 30 min in a −20 °C refrigerator. Following centrifugation at 12,000 rpm for 3 min, the supernatant was aspirated, and the sample was filtered through a microporous membrane with a pore size of 0.22 μm, then stored in an injection vial for UPLC-MS/MS analysis [22].

LC-MS parameters: column, Waters ACQUITY Premier HSS T3 (100 mm × 2.1 mm I.D., 1.8 μm, Waters, Milford, CT, USA); column temperature, 40 °C; mobile phase A comprising 0.1% formic acid in water; mobile phase B comprising 0.1% formic acid in acetonitrile at a flow rate of 0.4 mL/min. Separation was executed with a gradient elution protocol as follows: the initial composition was 95% A and 5% B, transitioning to 80% A and 20% B over 2 min, then to 40% A and 60% B over 3 min, followed by 1% A and 99% B for 1 min, maintained for 1.5 min, and finally reverting to 95% A and 5% B for 0.1 min, sustained for 2.4 min. The flow rate, column temperature, and injection volume were 0.4 mL/min, 40 °C, and 4.0 μL, respectively. The samples were detected in electrospray ionization (ESI) positive and negative ion modes with an ESI spray voltage of 5 kV.

### 2.5. Statistical Analysis

Data are expressed as mean ± standard deviation (SD) (*n* = 3). Variance (ANOVA) and Duncan’s multiple extreme variance test were performed using SPSS 20.0 (SPSS Science, Chicago, IL, USA). Comparisons between groups of homogeneous samples were performed using a *t*-test with a test level of 95%. Moreover, heatmap, k-means plot, heatmap + VIP plot, KEGG bubble plot, KEGG metabolite statistic plot were plotted using open source data analysis website (https://cloud.metware.cn/ accessed on 12 August 2024); KEGG metabolic pathway map using open source data website (https://www.figdraw.com/ accessed on 14 August 2024); PCA plot was made using open source data website (https://www.omicstudio.cn/ accessed on 16 August 2024); pie chart was made using Origin 2021 (64-bit).

## 3. Results

### 3.1. Physicochemical Characteristics of PAMJ

**Figure 1:**
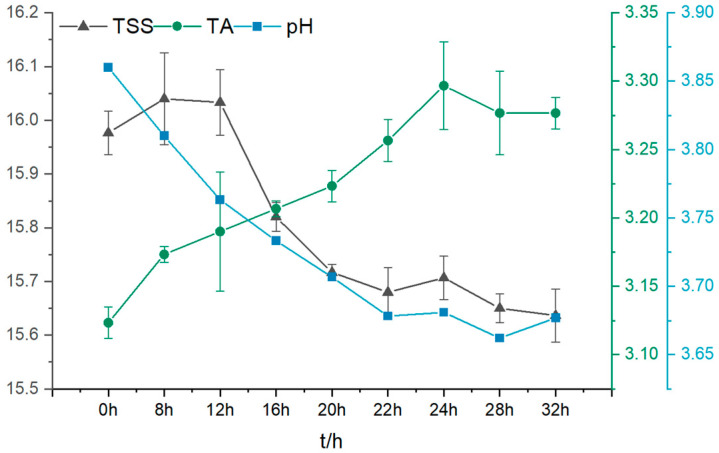
pH, TSS, and TA, of PAMJ at different fermentation times.

Figure 1 illustrates that during the fermentation by LAB, with the growth and multiplication of LAB, the LAB metabolized using the sugars in the juice to produce organic acids such as lactic acid, resulting in a gradual decrease in pH value. In contrast, the total acid content gradually increased, which is in agreement with the results of Xin An et al. [23]. A decreasing trend in soluble solids content was also observed throughout the fermentation process, mainly due to the consumption of sugars in the juice by the LAB during growth and fermentation. We can see that the decrease in pH is correlated with the increase in total acid content, reflecting the enhanced metabolic activity of LAB. The production of lactic acid during this process not only changes the juice’s acidity but may also affect its flavor and organoleptic properties. In addition, with the reduction in soluble solids, the sweetness and mouthfeel of the juice may be affected, thus having a significant impact on the acceptability of the final product.

### 3.2. Preliminary Identification of the Volatile Components of PAMJ

**Figure 2:**
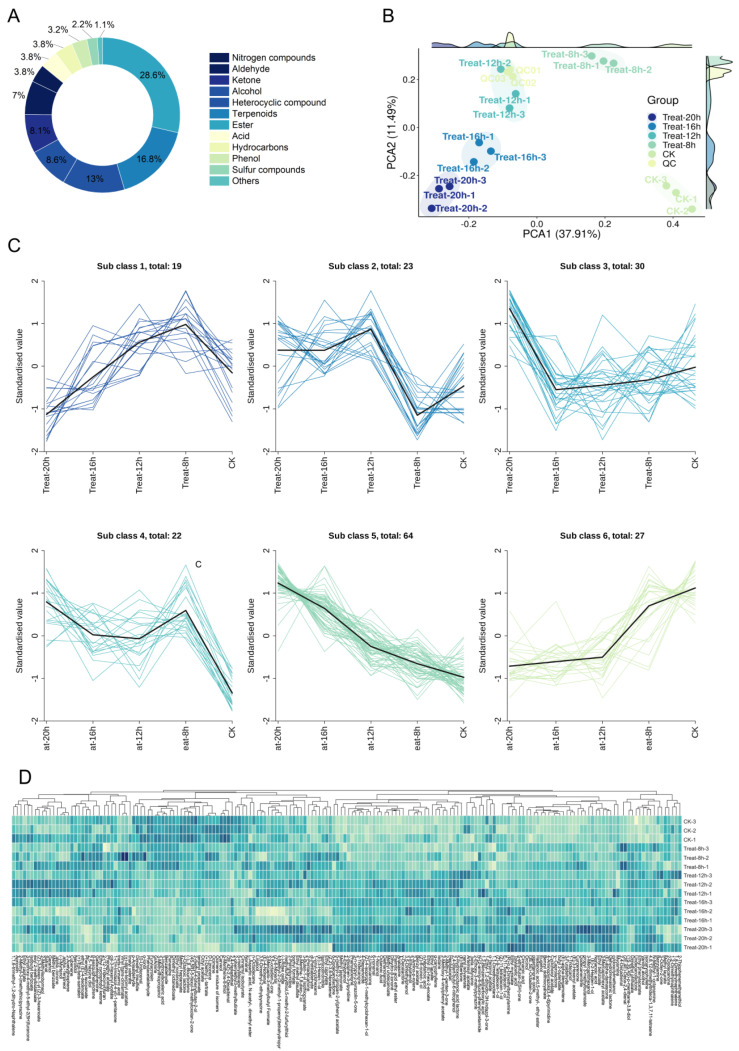
(**A**) Classification of volatile metabolites from PAMJ; (**B**) partial least squares analysis of volatile metabolite variations during lactic fermentation; (**C**) k-means plot of differential metabolites throughout lactic fermentation; (**D**) clustered heat map of volatile metabolites during lactic fermentation.

In order to elucidate the characteristics of flavor changes of PAMJ, we analyzed the volatile compound compositions of PAMJ during the lactic fermentation process. A total of 185 volatile compounds (Table S1) were screened according to VIP > 1, *p* < 0.05. In this study, 185 volatile compounds (Table S1) were categorized into terpenoids (31 species), ester (53 species), heterocyclic compounds (24 species), ketone (15 species), alcohols (16 species), aldehydes (13 species), hydrocarbons (7 species), acids (7 species), nitrogen compounds (7 species), phenols (6 species), sulfur compounds (4 species), others (2 species) (Figure 2A). Figure 2B illustrates the sample distribution derived using PCA. The first principal component (PC1) represents 37.91% of the total variance in the data, whereas the second principal component (PC2) represents 11.49%. Samples from the late lactic fermentation phase (Treat 16–20 h) were predominantly grouped in the lower left quadrant of the picture. In contrast, the 0 h fermentation period (CK) samples were concentrated in the lower right quadrant. These observations suggest that the volatile compound composition of the samples varied significantly between fermentation time points. We conducted a k-means cluster analysis on the 185 volatile compounds (Figure 2C), with the horizontal axis representing various lactic fermentation durations and the vertical axis indicating the concentrations of volatile compounds. In this study, six clusters were identified. Clusters 1 and 6 showed a decreasing trend in the content of volatile compounds after lactic fermentation and had a high percentage of terpenes and esters and a relatively high percentage of aldehydes. Clusters 2 and 4 showed fluctuating concentrations of volatile compounds during lactic fermentation and again had a high proportion of terpenoid esters and a relatively high proportion of heterocyclic compounds. A significant rise in the concentration of compounds in clusters 3 and 5 was detected post-fermentation, with the highest proportion of esters, and relatively high proportions of heterocyclic compounds, terpenoids, and ketones, and the next highest proportion of alcohols. Regarding fermentation time, it was observed that the content of volatile compounds in the six clusters significantly increased or decreased from 0 to 8 h. At the 8 to 16 h stage, on the other hand, the concentration of volatile compounds showed varying degrees of fluctuation (i.e., increased or decreased). In contrast, at the 16 to 20 h stage, the concentrations of volatile compounds in all clusters showed a single trend, showing a significant increase or decrease. The results demonstrate that the dynamic change characteristics of volatile compounds and their compositional differences in different clusters during lactic fermentation provide an important basis for further research on biotransformation during lactic fermentation.

### 3.3. Dynamics of Volatiles in PAMJ During the Lactic Fermentation Process

Figure 2D illustrates the variation of particular volatile compounds throughout lactic fermentation at different periods. Table S2 has detailed information and data. To clarify the dynamic alteration of volatiles during PAMJ lactic fermentation, samples collected at 0, 8, 12, 16, and 20 h of fermentation were examined (Table S2). The results indicated that the number of differential metabolites exhibited an initial increase followed by a subsequent decrease as lactic fermentation advanced. During the initial phase of fermentation (0–8 h), the content of phenolic compounds increased significantly, with the most significant increase in the eugenol content, which reached 15.20 μg/L (Table S2). Meanwhile, the content of terpenoids was significantly down-regulated, as evidenced by a decrease of 3.78 μg/L for D-limonene (Table S2) and a decrease of 4.38 μg/L for ocimene and its mixture of isomers (Table S2). In addition, the generation of the new terpene ocaryophyllene conferred a woody spice flavor to the samples. Eugenol [24] and D-limonene [25] are the main flavor substances in apricot, imparting strong lemon, citrus, and floral-fruity flavors to apricot, while the latter imparts a floral flavor to peaches. The primary process for producing eugenol is the phenylalanine metabolic pathway [26], and monoglucosides are typically found as its precursors [27]. Thus, at the beginning of fermentation, eugenol may be present mainly in its precursor form. As fermentation proceeds, microorganisms release eugenol from the sugar-fixation precursors through enzymatic degradation, resulting in an increased concentration of eugenol [27]. This phenomenon is consistent with the findings of Yuhan Yuan et al. [28] who found in their experiments with fermented peach juice that the content of eugenol, a key component, increased 14.27 times at the end of fermentation. This suggests that the metabolic activity of LAB promotes the conversion of sugars and significantly elevates the concentration of eugenol, affecting the flavor and chemical composition of fermented juices. In contrast, the total terpenoid concentration decreased as lactic fermentation progressed, which may be explained by the progressive breakdown of terpenoids into free aroma compounds due to glycosidases at work [24].

During the lactic mid-fermentation period (8–16 h), the content of phenolic compounds continued to increase, especially the concentrations of 3-ethylphenol (16.91 μg/L), 3,5-dimethylphenol (16.91 μg/L), and eugenol (7.40 μg/L) increased significantly (Table S2). Meanwhile, the content of the alcohol compound 3-methylthiopropanol decreased by 41.07 μg/L, and the content of benzaldehyde decreased by 37.09 μg/L (Table S2). In addition, two new ketones were generated at this stage: (E)-filbertone and (E)-alpha-damascone enriched the aroma hierarchy. However, the content of eugenol continued to increase, the overall content of volatile substances decreased, and the floral and fruity aromas were weakened. Changes in volatile compounds at this stage showed a more complex dynamic profile. The concentration of phenolic compounds rose during the midpoint of lactic fermentation, particularly the level of eugenol, which was in stark contrast to the notable decline in alcohol content. This phenomenon is consistent with the results of existing studies, such as Niu et al. [29]. Notably, a negative correlation was identified between the levels of 3-methylthiopropanol and eugenol. Consequently, the substantial reduction in alcohol content (3-methylthiopropanol) during the mid-phase of lactic fermentation may have facilitated the elevation of eugenol and other phenolic compounds. In addition, alcohols act as precursors to esters and usually provide floral and fruity aromas. Nevertheless, the concentration of these compounds diminished during lactic fermentation, likely due to their consumption in esterification processes to produce the corresponding esters [4]. During the early stages of Lactobacillus fermentation, benzaldehyde in PAMJ exhibited a slight increase, which may be related to the oxidation of fatty acids and the metabolism of amino acids. However, in the middle stage of fermentation, the content of benzaldehyde decreased significantly. Related studies have shown that when apricot juice is fermented using Saccharomyces cerevisiae, the content of aldehydes also decreases in the later stages of fermentation, which is consistent with the results of this study [30]. However, the study by Yang et al. [14] showed a significant increase in the content of benzaldehyde at the later stages of fermentation. The decrease in benzaldehyde content in this experiment may be attributed to the instability of aldehydes, which can be easily reduced to alcohols or oxidized to acids in food matrices, leading to a decrease in the aldehyde content at later stages of fermentation. Finally, aldehydes had relatively little effect on the flavor of the PAMJ compared to other VOCs due to their higher odor detection threshold. Nonetheless, benzaldehyde remained a significant component of almond aroma [14], indicating its ongoing contribution to flavor profile development despite its diminished concentration.

During the advanced phases of lactic fermentation (16–20 h), the classes of volatiles remained the same, but the content of each volatile substance decreased (Table S3). The manifestations of floral and fruity flavors diminished to varied extents during this phase, perhaps due to a decrease in terpene and ester levels. This phenomenon is similar to the findings of Aihmaitijiang et al., who also showed a decrease in the types of volatile compounds in the later stages of fermentation [31]. The eugenol content grew by 16.92 μg/L during lactic fermentation (Table S2). However, the rate of rise of eugenol content markedly diminished towards the conclusion of the lactic fermentation compared to the middle phase. In the meantime, there was a reduction of 8.06 μg/L in γ-decalactone concentration. After lactic fermentation, the concentration of γ-decalactone, responsible for the distinctive flavor of peaches and apricots, decreases, probably because the volatilization or decomposition of this substance exceeds the rate of its production [32]. Furthermore, as the lactic fermentation advances into later phases, the number of viable bacteria stays comparatively constant, the fermentation process stabilizes, the pace at which proliferation and death converge simultaneously, and the amount of organic acids produced stabilizes. This series of alterations may also result in a deceleration of the production and accumulation rate of flavor compounds, thereby causing a reduction in the concentration of flavor constituents. On the other hand, the depletion of nutrients, the decline in pH value, and other factors may intensify the competition between different species of *Lactobacillus*, resulting in the reduction or disappearance of certain species of Lactobacillus, which will hurt the formation of flavor production [33].

### 3.4. Analysis of rOAV During the Lactic Fermentation Process

**Figure 3:**
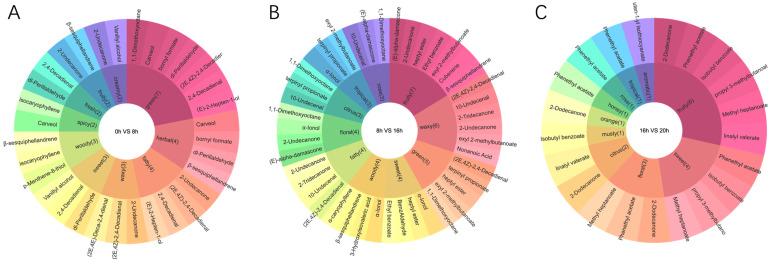
Flavor wheels of differentially accumulated metabolites: (**A**) flavor wheel of differentially accumulated metabolites in the pre-fermentation stage (0–8 h); (**B**) flavor wheel of differentially accumulated metabolites in the mid-fermentation stage (8–16 h); (**C**) flavor wheel of differentially accumulated metabolites in the late fermentation stage (16–20 h). Inner circles, different stages of fermentation; middle circles, associated odor descriptions; outer circles, names of differential metabolites with associated flavors.

In lactic fermentation, key aroma components and relative odor activity value (rOAV) demonstrated the role of aroma constituents in the food aroma system. The threshold value denotes the minimum concentration of an aroma chemical at which humans initially perceive an aroma. The relative odor activity value (rOAV) is determined by dividing the relative concentration of each flavor component by its odor threshold [34]. Typically, an rOAV ≥ 1 signifies that the compound directly influences the flavor of the sample [4,34]. Although the odor thresholds reported in different research may differ, comparing rOAV levels of PAMJ at different lactic fermentation stages improved the final product’s unique flavor. Sensory analysis of differential metabolites can be utilized further to understand their contribution to the overall taste profile. Based on the rOAV of the differential metabolites, we investigated the effect of differential metabolites on flavor (Figure 3). The flavor profile showed significant variations during the different stages of lactic fermentation. The most prominent flavors in the lactic pre-fermentation phase included green, fat, and herbal. As lactic fermentation progressed into the middle stages, fruity, waxy, and green flavors began to dominate (Table S3). Eventually, in the later stages of lactic fermentation, fruity, sweet, and floral flavors became the most prominent flavor profiles. This process of change reflects the transformation of chemical components during lactic fermentation and its effect on flavor.

### 3.5. Changes in Non-Volatile Compounds During Lactic Fermentation

**Figure 4:**
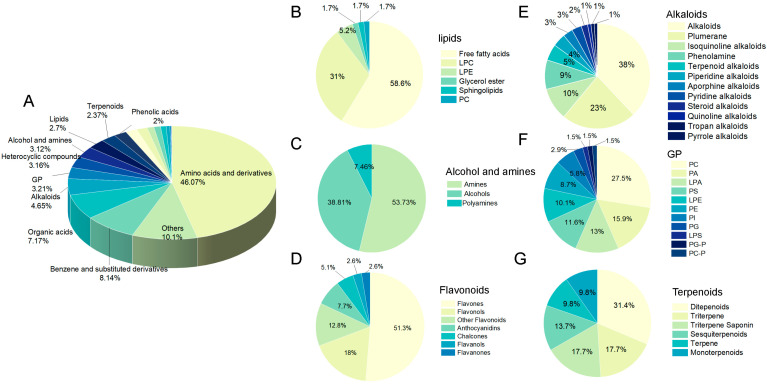
(**A**) Pie chart illustrating the primary classification of non-volatile compounds identified by UHPLC-MS/MS; (**B**–**G**) pie chart depicting the secondary classification within the first classification.

Klee [35] identified that the precursors of aroma compounds predominantly consisted of amino acids, carotenoids, fatty acids, and other essential nutrients. To gain a deeper understanding of the specific alterations in flavor, we also explored the changes in the composition of peach and PAMJ before and after lactic fermentation at the level of nonvolatile metabolites. We applied UHPLC-MS/MS to evaluate the metabolic characteristics of PAMJ before and after lactic fermentation. 6292 non-volatile metabolites were discovered in both positive and negative ion modes, including 2458 amino acids and their derivatives. 549 benzene and their derivatives, 539 organic acids, 288 alkaloids, 173 glycerophospholipids, 180 heterocyclic compounds, 249 alcohols and amines, 448 lipids and lactones, 181 terpenes, 172 phenolic acids, 156 flavonoids, 125 sugars, and 156 flavonoids. 156 kinds, 125 kinds of sugars, 101 kinds of nucleotides and their derivatives, 109 kinds of fatty acyls, 74 kinds of lignans and coumarins, 63 kinds of glycerol lipids, 28 kinds of steroids, 12 kinds of sphingolipids, 11 kinds of quinones, 9 kinds of tryptophan, choline, and pigments, 9 kinds of tannins, and 358 kinds of others. The proportions of the components of PAMJ are shown in Figure 4.

PAMJ was initially differentiated at the beginning and conclusion of lactic fermentation phases using principal component analysis (PCA) (Figure S1). The two primary components represented roughly 62.29% of the total variation, with PC1 contributing 56.94% and PC2 12.35%, respectively. The PCA score plots demonstrated a clear separation between the initial and final stages of lactic fermentation, implying a difference between the PAMJ samples from the two phases. VIP > 1, *p*-value < 0.05 (*T*-test), and absolute Log2FC (fold change) >2 or <0.5 were used to screen for differentiated metabolites. During lactic fermentation, 1846 compounds were discovered, with 564 up-regulated and 1282 down-regulated (Table S4). Among these differential metabolites, organic acids, amino acids, and derivatives were primarily accumulated via LAB metabolism, whereas organic oxygenates, lipids, and lipid-like molecules were primarily consumed during lactic fermentation, indicating that carbohydrates, some amino acids, and lipids were the primary precursors [36]. In addition, lignin, nucleotides, and other substances accumulate due to chemical reactions, possibly also due to the degradation of amino acids through the melodic reaction.

#### 3.5.1. Amino Acids and Peptides

**Figure 5:**
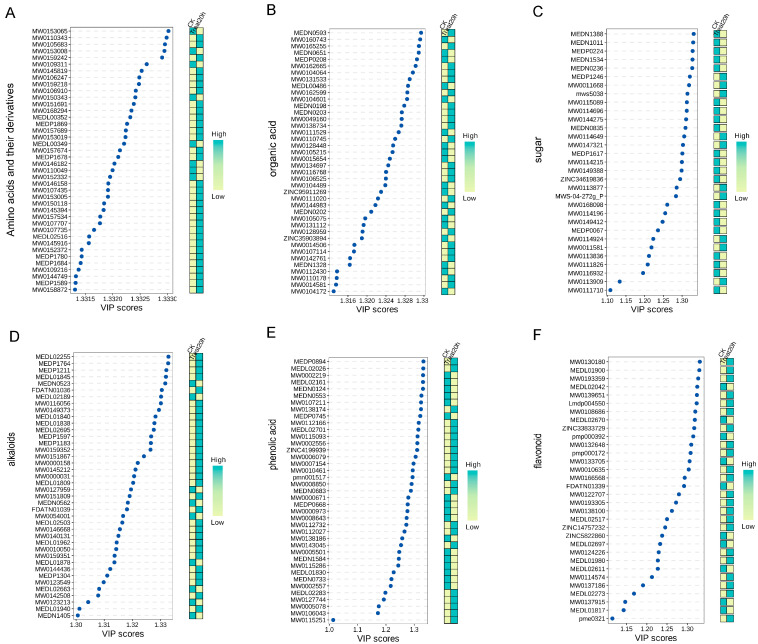
UHPLC-MS/MS to obtain non-volatile compounds differing significantly in PAMJ: (**A**) Heatmap of the top 40 amino acid and peptide compounds with significant differences + VIP; (**B**) Heatmap of the top 40 sugar compounds with significant differences + VIP; (**C**) Heatmap of the top 40 organic acid compounds with significant differences + VIP; Heatmap of the top 40 alkaloids with significant differences + VIP; (**D**) Heatmap of the top 40 phenolic acids with significant differences + VIP; (**E**) Heatmap of the top 40 phenolic acids with significant differences + VIP; (**F**) Heatmaps of the top 40 alkaloids with significant differences + VIP. Flavonoids with significant differences.

Amino acids are a class of flavor-active compounds classified as sweet, fresh, and bitter amino acids based on their sensory properties [37]. Amino acids play a crucial role in the control of human health. Amino acids are key precursors of taste compounds such as esters, higher alcohols, and pyrazines, which might influence the scent of the samples [38]. This study used thermograms and VIP plots to assess the top 40 amino acids, peptides, and analogs that exhibited substantial modifications (Figure 5A) (Table S3 for detailed data). After lactic fermentation, 32 amino acids and peptide substances showed a down-regulation trend, including high arginine, L-isoleucine-L-tyrosine, glutamic acid-valine, and isoleucine-tryptophan. LAB utilized resources, including amino acids and proteins, in PAMJ to facilitate their growth and metabolic functions [39], reducing total amino acids and peptide substances in PAMJ following the completion of lactic fermentation. The results showed a significant increase in eight amino acids and peptide substances, including tryptophan-alanine-arginine, Asp-Lys-Arg-Glu-Lys, L-leucine-L-glutamic acid-L-valine, and serine-leucine with multiples of 29.59 times, 11.39 times, and 9.10 times, respectively (Table S4). Among the detected amino acids and peptide substances, the number of peptides was found to be much higher than the number of free amino acids, most likely because LAB was able to break down the proteins in the juice into smaller peptide fragments and a small amount of small-molecule amino acids through the action of fermented fruit during the lactic fermentation process [40]. Research indicates peptides possess buffering capacity and can contribute delicate and subtle flavors to foods. The flavor attributes of the constituent amino acids predominantly determine the flavor characteristics of peptides. Amino acids can be metabolized into the intermediate metabolite pyruvic acid, which is converted directly or indirectly into the volatile compound acetaldehyde, effectively enhancing the flavor of PAMJ in this way [41].

#### 3.5.2. Sugars and Organic Acids

The predominant flavors of flat peach and white apricot are characterized by sourness and sweetness, and organic acids, including citric, succinic, and malic acids mainly influence their acidity. After the lactic fermentation was completed, the content of isomaltose and alpha-heptasaccharide in the PAMJ decreased significantly, indicating that the LAB utilized the carbohydrates in the mixed PAMJ to provide energy for the microorganisms and entered the TCA cycle via the glycolytic pathway to generate organic acids, alcohols, aromatic amino acids, and phenolic compounds [42,43]. The concentrations of D-glucoseheptose, 1-deoxy-D-xylulose, L-gulose, mannitol, and cottonseed sugar were markedly elevated. Among them, mannitol is a natural hexitol converted from fructose. This investigation revealed significant amounts of mannitol in the fermented PAMJ, highlighting the essential role of LAB in mannitol production, which enhances the PAMJ’s agreeable sweetness. In addition, the study by Bingsen Feng et al. [44] also found that the content of mannitol increased significantly during the fermentation of peach, which is consistent with our findings.

Organic acids are vital in plants’ primary and secondary metabolism, and the appropriate proportion of them lends a fresh and crisp flavor. Furthermore, they increase polysaccharide conversion and pectin component breakdown, enabling juice fermentation and clarity. Some organic acids are formed during the metabolism of amino acids. For example, in LAB strains such as *L*. *plantarum* and *L*. *brevis*, the transamination of leucine yields 4-methyl-2-oxovaleric acid (KICA), which is then reduced to A-hydroxyisohexanoic acid. Thus, the rise in organic acid concentration could result from juice fermentation by *L*. *plantarum* and *L*. *brevis* [45]. The concentrations of succinic acid, all-trans-heptaprenyl diphosphate, 2-amino-3-(1H-pyrazol-1-yl)propanoic acid, hydroxyisocaproic acid, 3-(3-hydroxyphenyl) propionic acid, 4-oxoretinoic acid, and mandelic acid increased dramatically at the end of fermentation. Succinic acid is produced under anaerobic conditions from malic acid or the degradation of specific amino acids. The acidity of the fermentation broth affects the yield of succinic acid, which enhances the acidity of PAMJ and generates esters. Additionally, succinic acid is a crucial substrate in the TCA cycle, facilitating the degradation of lactic acid, alleviating fatigue, and exhibiting anti-aging, appetite-stimulating, and disease-preventive attributes [46].

#### 3.5.3. Alkaloids

Alkaloids are nitrogenous alkaline compounds, several of which taste bitter [47]. Forty significantly altered alkaloids were detected in PAMJ, which were categorized into seven subclasses, including alkaloids (13), plumeranes (9), phenolamine (6), isoquinoline alkaloids (3), terpenoid alkaloids (2), aporphine alkaloids (2), piperidine alkaloids (1), and others (4). After lactic fermentation, the concentration of nine of these alkaloids significantly rose, with indole-3-lactic acid rising by 1299.94 times. Indole-3-lactic acid is a natural flavor enhancer that provides freshness components such as glutamate, inosinic acid, and guanosinic acid, which make up the freshness of food. In addition, indole-3-lactic acid contains a complex flavor profile of many amino acids, organic acids, peptides, and sugars, giving PAMJ a rich, mellow flavor and the ability to mellow out harsh tastes.

#### 3.5.4. Polyphenols

Polyphenols are significant secondary metabolites characterized by bitter and astringent tastes. Increasing data indicate that polyphenol-rich products may positively impact non-communicable diseases by improving hypertension, hyperlipidemia, hyperglycemia, and oxidative stress [48]. This study identified 38 phenolics, 28 flavonoids, and one proanthocyanidin with significant differences. By 44.69 times, 21.79 times, 5.73 times, and 4.15 times, respectively, the quantities of m-cresol, p-coumaryl alcohol, proanthocyanidins, and cianidanol increased significantly at the end of lactic fermentation. The augmentation of total flavonoid content may result from the transformation of complicated flavonoid glycosides into various simple flavonoids during the lactic fermentation of PAMJ. A correlation may exist between the rise in phenolic acid content and the activity of phenylalanine ammonia-lyase during the intermediate and final stages of lactic fermentation [49]. Lactic fermentation significantly improved the PAMJ’s bioavailability, nutritional efficacy, and flavor profile [50,51].

#### 3.5.5. Analysis of Key Metabolic Pathways

**Figure 6:**
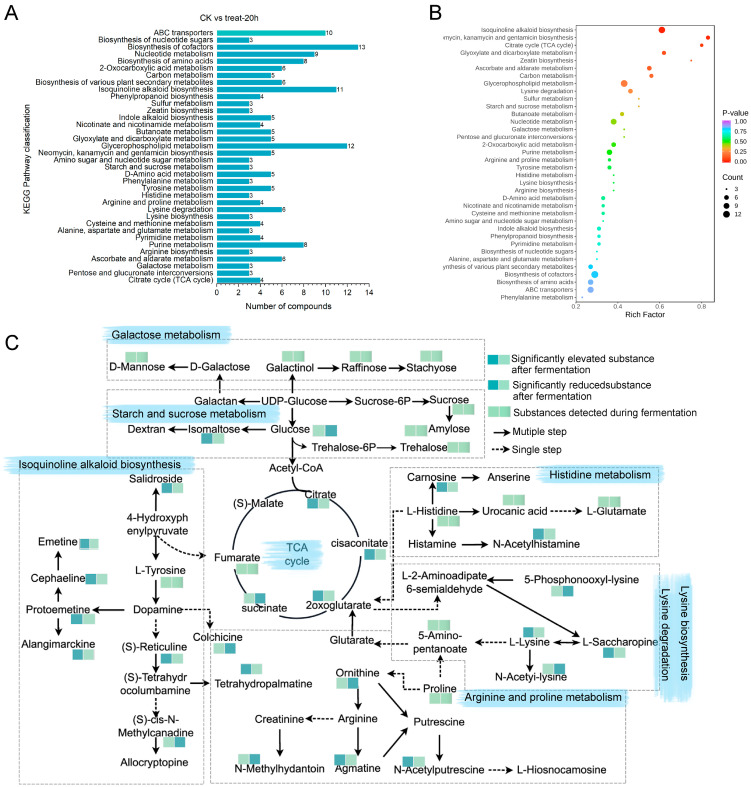
(**A**) KEGG topological analysis; (**B**) KEGG metabolite statistics; (**C**) KEGG-based pathway module associations of principal differential metabolites of PAMJ flavors; red and green substances represent differential metabolites within the metabolic pathway, blue substances denote metabolites identified in the metabolic pathway with insignificant alterations pre- and post-fermentation, while black substances signify intermediate metabolites in the metabolic pathway.

It is inadequate to base analysis only on the kind and concentration of these compounds because fermentation is a complicated metabolic process that produces flavor substances and bioactive components. Pathway analysis can help uncover important metabolites and their material regulation mechanisms. This study aimed to look at the important metabolic pathways involved with the differences in metabolites before and after lactic fermentation of PAMJ. By comparing the results with the KEGG database, we identified a total of 38 key metabolic pathways before and after lactic fermentation, including isoquinoline alkaloid biosynthesis (*p* < 0.05), neomycin, kanamycin and gentamicin biosynthesis (*p* < 0.05), and TCA cycle (*p* < 0.05) showed significant enrichment (Figure 6A). The combined analysis of *p*-value, enrichment factor, and DA-scores indicated that the expression of the citric acid cycle and isoquinoline alkaloid biosynthesis significantly influenced the creation of unique flavor profile compounds in the PAMJ. During lactic fermentation, as shown in Figure 6B, combined with metabolomics data, we identified the metabolic pathways linked to flavor based on the quantity of differentially accumulated metabolites and the alterations in there. The primary metabolic pathways that affect the number of diverse metabolites during lactic fermentation and their conversion relationships are as follows: in the saccharification process, polysaccharides in PAMJ are initially converted to glucose through the glycolytic pathway before entering the TCA cycle. As the major hub of sugar, amino acid, and lipid metabolism, the TCA cycle has a considerable impact on food flavor, mostly via modulating intermediate and enzyme activity in metabolic pathways. Organic acids, such as oxaloacetic acid, enter the TCA cycle via acetyl-coA to make citric acid [23], which is metabolized to generate unique scents and flavors that directly influence food taste. For example, dehydrogenation and decarboxylation of isocitrate acid produce 2-oxoglutarate, which is converted to succinyl-CoA through oxidative decarboxylation and ultimately to succinic acid. In addition, succinic acid is produced in the TCA cycle and further converted to various metabolites such as arginine, proline, and glutamate. The metabolic pathway in Figure 6C shows that the TCA cycle is closely related to the synthesis and degradation of lysine, which has diverse flavor characteristics, including sweet, sour, fresh, salty, and bitter, which confer different flavor characteristics to foods. Therefore, the degradation of lysine affects its flavor contribution and further alters the overall flavor of foods by influencing the interactions with other flavor substances. Furthermore, histidine, 2-oxoglutarate, and cisaconitic acid are significant metabolic substrates in the formation of PAMJ. The anticipated metabolic pathways align with alterations in the relative quantity of distinct metabolites associated with the PAMJ seen throughout the synthesis.

These findings indicate that the flavor created by PAMJ is directly tied to alterations in these key metabolites. Metabolic profiles modified following lactic fermentation and matrix effects may impact component quantification using LC-MS. To reduce matrix effects, future research should focus on metabolite analysis in conjunction with optimal sample pretreatment approaches, isotope labeling, and combinations of LC-MS/MS methods to alleviate matrix effects. More research is required to clarify the integrated biotransformation pathways related to these compounds.

## 4. Conclusions

This study employed flavor analysis and non-targeted metabolomics to investigate the specific changes in flavor compounds during the mixed lactic fermentation of PAMJ with five Lactobacillus species, alongside the metabolic variations of non-volatile compounds pre-fermentation and post-fermentation, revealing that the flavor differences were linked to the accumulation of specific metabolites. A total of 185 significant volatile compounds and 1846 prominent non-volatile chemicals were successfully identified during the study. The principal differential metabolites of PAMJ at various lactic fermentation duration times have been identified as eugenol, 3-methylthiopropanol, and benzaldehyde. Flavor wheel analysis revealed that the fermentation of PAMJ resulted in the enrichment of volatile compounds, predominantly characterized by fruity, sweet, and floral notes, with (E)-alpha-damascone, phenethyl acetate, ethyl benzoate, isobutyl benzoate, and 2-undecanone as the primary contributors. The non-targeted metabolomics analysis identified 2-oxoglutarate, succinic acid, citrate hydrochloride, lysine, and ornithine as significant differential metabolites. Furthermore, phenolic contents were elevated with an extended lactic fermentation period, signifying enhanced antioxidant capabilities of PAMJ. Through untargeted metabolomics, we identified starch and sugar metabolism, amino acid metabolism, and TCA cycling as metabolic pathways significantly influencing flavor before and following PAMJ lactic fermentation. Our findings indicated that various lactic fermentations of PAMJ significantly influenced flavor and metabolites, suggesting that the five LAB strains utilized successfully increased PAMJ’s flavor. Variations among strains may enhance the development and innovation of PAMJ processing techniques and other associated plant-based food bioprocessing processes.

## Data Availability

The original contributions presented in the study are included in the article/Supplementary Materials; further inquiries can be directed to the corresponding author.

## Supplementary Materials

The following supporting information can be downloaded at: https://www.mdpi.com/article/10.3390/foods13233835/s1, Figure S1: partial least squares analysis of non-volatile metabolite variations during fermentation; Table S1: 185 volatile compound components in PAMJ during lactic fermentation; Table S2: volatile compounds screened by PAMJ under GC-MS conditions for different fermentation times; Table S3: relative odor activity values (rOAV ≥ 1) of volatile compounds during lactic fermentation of PAMJ; Table S4: 1846 non-volatile compound components in PAMJ during fermentation.
