# Supplementary material for: Study on the Mechanisms of Flavor Compound Changes During the Lactic Fermentation Process of Peach and Apricot Mixed Juice
Source: Foods. 2024 Nov 28;13(23):3835. doi: 10.3390/foods13233835 (PMC11639762; doi:10.3390/foods13233835)
Supplement: Supplementary file 1 [file foods-13-03835-s001.zip › Table S1;Table S3;Figure S1.pdf]

Table S1. 185 Volatile Compound Components in PACJ during Fermentation

No.	compound	CAS	NR	Cocentrantion (μg/L)				
				CK	Treat-8	Treat-12	Treat-16	Treat-20
Alcohol								
1	2-methyl-4-phenylbutan-2-ol	103-05-9	1299.41	0.33±0.04a	0.2±0.03b	0.21±0.01b	0.17±0.02b	0.12±0.02c
2	1-Tridecanol	112-70-9	1577.00	0.34±0c	0.54±0.07b	0.48±0.01b	0.56±0.02b	0.69±0.09a
3	1-Nonanol	143-08-8	1170.64	3.77±0.16a	4.26±0.37b	5.53±0.4b	5.56±0.26c	6.32±0.33c
4	1-vinyl-cyclohexano	1940-19-8	1020.00	0.03±0b	0.42±0.1a	0.37±0.12a	0.28±0.06ab	0.47±0.27a
5	α-Ionol	25312-34-9	1455.00	0.12±0.07a	0.13±0.11a	0.06±0.01a	0.04±0a	0.05±0a
6	2,5- hexyne diol	3031-66-1	960.00	0.05±0.01a	0.05±0.01a	0.04±0.01a	0.04±0.01a	0.01±0.01b
7	(Z)-6-nonen-1-ol	31502-19-9	1167.00	0.07±0.06b	0.15±0.02b	0.3±0.07a	0.31±0.08a	0.32±0.02a
8	（E)-2-Hepten-1-ol	33467-76-4	978.00	0.03±0.01a	0.01±0.01a	0.07±0.05a	0.05±0.09a	0.12±0.11a
9	(Z)-11-tetradecen-1-ol	34010-15-6	1678.00	0.1±0.07b	0.11±0.1b	0.14±0.05b	0.3±0.03a	0.27±0.04a
10	(Z)-9-Tetradecen-1-ol	35153-15-2	1666.00	0.1±0.07b	0.11±0.1b	0.14±0.05b	0.3±0.03a	0.27±0.04a
11	E-11-Tetradecen-1-ol	35153-18-5	1673.00	0.1±0.07b	0.11±0.1b	0.14±0.05b	0.3±0.03a	0.27±0.04a
12	cyclooctylmethanol	3637-63-6	1073.00	0.25±0.04a	0.34±0.15a	0.63±0.57a	0.45±0.38a	0.78±0.31a
13	Vanillyl alcohol	498-00-0	1423.00	0.01±0c	0.04±0.03bc	0.06±0.02ab	0.06±0.02ab	0.08±0.01a
14	3-Methylthiopropanol	505-10-2	978.28	53.68±8.28a	50.78±2.52a	24.97±6.29b	9.71±2.7c	7.88±0.89c
15	3-Mercaptohexanol	51755-83-0	1127.00	0.11±0.01c	0.14±0bc	0.16±0.01bc	0.17±0.02b	0.23±0.06a
16	(E)-3-Hexen-1-ol	928-97-2	852.00	5.16±0.98a	6.64±1.36a	6.06±0.77a	5.01±0.53a	6.41±1.73a
Phenol								
17	3-Ethylphenol	620-17-7	1169.00	9.09±5.61b	9.25±3.59b	22.36±6.18ab	26.16±12.63a	23.31±2.49a
18	Eugenol	97-53-0	1362.36	46.96±5.22c	62.17±2.78b	58.36±2.18bc	69.57±4.63b	86.49±14.99a

19	4-Ethylphenol	123-07-9	1165.40	0.27±0.21b	0.38±0.21ab	0.53±0.05ab	0.58±0.05a	0.65±0.04a
20	Orcinol	504-15-4	1368.25	0.12±0.02c	0.15±0.02bc	0.17±0.03b	0.19±0.01b	0.24±0.01a
21	3,5-Dimethylphenol	108-68-9	1171.00	9.09±5.61b	9.25±3.59b	22.36±6.18ab	26.16±12.63a	23.31±2.49a
22	2,4-ditert-butyl-6-nitrophenol	20039-94-5	1688.00	0.05±0.01c	0.07±0c	0.54±0.02a	0.3±0.04b	0.29±0.09b
Nitrogen compounds								
23	Benzylamine	100-46-9	1013.99	0.05±0.05a	0.08±0.06a	0.12±0.04a	0.12±0.04a	0.09±0.07a
24	2-Amino-p-xylene	95-78-3	1171.00	0.72±0.4b	0.76±0.2b	1.91±0.35a	1.81±0.44a	2.02±0.24a
25	Diethanolamine	111-42-2	1349.00	0.25±0.04d	0.31±0.02cd	0.37±0.05bc	0.42±0.02ab	0.5±0.09a
26	N-Methyl-1-octanamine	2439-54-5	1088.00	0.45±0.02b	2.03±2.76ab	3.24±0.17a	2.55±0.04ab	3.22±1.27a
27	(R)-α,p-Dimethylbenzylamine	4187-38-6	1185.00	0.03±0.03a	0.04±0.03a	0.06±0.04a	0.18±0.12a	0.18±0.12a
28	N-(2-amino-6-fluoro-phenyl)acetamide	8645-85-7	1580.00	0.02±0b	0.07±0.04ab	0.09±0.04a	0.03±0.01ab	0.07±0.05ab
29	Dodecanenitrile	2437-25-4	1490.00	2.11±0.13b	2.24±0.18ab	2.18±0.13ab	2.4±0.13a	2.42±0.1a
30	Dimethyl trisulfide	3658-80-8	972.31	0.12±0.03a	0.09±0.05ab	0.06±0.02ab	0.03±0.01b	0.07±0.03ab
31	p-Menthene-8-thiol	71159-90-5	1283.00	0.57±0.05a	0.24±0.04b	0.27±0.02b	0.26±0.02b	0.3±0.04b
32	Benzyl isothiocyanate	622-78-6	1367.00	5.01±0.34c	7.07±0.18b	7.01±0.31b	8.43±0.4b	10.32±1.71a
33	Ethyl methyl disulfide	20333-39-5	846.00	0.23±0.04a	0.23±0.02a	0.26±0.04a	0.22±0.03a	0.27±0.07a
others								
34	1,1-Dimethoxyoctane	10022-28-3	1178.00	0.26±0.08a	0.1±0.11b	0.23±0.01a	0.22±0.01a	0.24±0.05a
35	3-Buten-1-yl Isothiocyanate	3386-97-8	983.00	0.12±0.08a	0.14±0.09a	0.13±0.17a	0.04±0a	0.16±0.1a
Aldehyde								

36	p-Anisaldehyde	123-11-5	1259.75	0.26±0.03a	0.26±0.04a	0.23±0.01ab	0.21±0.02b	0.22±0.02ab
37	2,4-Hexadienal	142-83-6	910.00	0.08±0.01a	0.05±0.01c	0.01±0.01b	0.07±0.02a	0.04±0.01b
38	Benzaldehyde	100-52-7	962.46	45.34±2.95a	45.62±2.71a	23.48±3.8b	8.53±1.23c	7.07±1.43c
39	Phenylacetaldehyde	122-78-1	1045.60	8.82±0.3a	7.03±1.2b	5.31±1.12bc	5.34±0.6bc	5.09±1.04c
40	Nonanal	124-19-6	1105.03	13.45±0.76a	13.37±0.11a	9.88±1.01b	10.39±0.57b	11.4±1.35b
41	(Z,Z)-3,6-Nonadienal	21944-83-2	1100.00	22.61±1.43a	24.11±1.68a	21.72±2.28a	21.65±0.43a	21.5±3.97a
42	dl-Perillaldehyde	2111-75-3	1274.00	0.03±0b	0.32±0.04a	0.23±0.17ab	0.24±0.05ab	0.26±0.19ab
43	(2E,4Z)-2,4-Decadienal	25152-83-4	1295.00	0.73±0.05a	0.22±0.03b	0.07±0.02c	0.07±0.02c	0.08±0.02c
44	(2E,4E)-Deca-2,4-dienal	25152-84-5	1320.08	0.06±0b	1.34±0.9a	0.49±0.04b	0.53±0.04b	0.56±0.02b
45	6-nonenal	2277-20-5	1124.00	0.02±0b	0.02±0b	0.16±0.05a	0.14±0a	0.18±0.06a
46	(E)-4-Nonenal	2277-16-9	1105.00	5.02±0.45a	5.01±0.75a	5.06±0.5a	4.93±0.1a	5.28±0.53a
47	(Z)-6-Nonenal	2277-19-2	1103.52	77.26±2.76a	76.48±5.1a	74.86±5.47a	73.73±0.53a	77.1±4.34a
48	10-Undecenal	112-45-8	1297.00	0.55±0.04a	0.43±0.18a	0.24±0.01b	0.22±0.01b	0.26±0.03b
Acid								
49	Nonanoic acid	112-05-0	1266.56	0.47±0.15c	0.49±0.32c	0.85±0.24bc	1.07±0.24b	1.61±0.04a
50	Octanoic Acid	124-07-2	1172.43	4.23±0.75b	4.86±0.26b	7.04±1.71a	7.29±1.08a	8.93±0.97a
51	Cinnamic acid	140-10-3	1451.00	0.02±0b	0.03±0.01ab	0.03±0ab	0.04±0.01a	0.03±0ab
52	Cinnamic acid	621-82-9	1427.53	0.16±0.01a	0.14±0.02a	0.16±0.01a	0.28±0.3a	0.54±0.36a
53	3-Hydroxyisovaleric acid	625-08-1	980.00	0.07±0.01a	0.07±0.01a	0.04±0.01b	0.01±0c	0.01±0c
54	n-heptanoic acid	111-14-8	1079.91	0.2±0.12b	0.09±0.05ab	0.28±0.14a	0.38±0.11a	0.37±0.03a
55	Tolylacetic acid	622-47-9	1363.00	1.46±0.13b	2.29±0.13ab	2.3±0.58ab	2.9±0.76b	4.07±0.86a
Terpenoids								
56	p-Mentha-1,8-dien-7-ol	536-59-4	1296.00	0.27±0.02ab	0.26±0.01b	0.25±0.02ab	0.25±0.01ab	0.29±0.04a

57	(+)-Epi-beta-santalen	25532-78-9	1448.00	0.26±0.01b	0.39±0.01a	0.38±0.03a	0.21±0.14bc	0.13±0.01c
58	(1S,3S,4S)-p-menthane-3,8-diol	3564-95-2	1355.00	0.45±0.04c	2.18±0.11a	2.1±0.06a	2.28±0.08a	0.89±0.16b
59	(Z)-4-isopropyl-1-methylcyclohexan-1-ol	3901-93-7	1167.00	0.24±0.19d	0.57±0.05c	0.94±0.07b	1.04±0.07b	1.22±0.03a
60	.beta.-Myrcene	123-35-3	992.02	14.18±3.03a	16.07±3.04a	17.08±2.69a	17.37±2.66a	19.39±4.57a
61	D-Limonene	5989-27-5	1031.27	39.2±4.39a	35.42±5.96a	32.14±6.12ab	32.51±1.04ab	26.93±1.13b
62	Ocimene mixture of isomers	3338-55-4	1038.00	39.89±4.35a	35.51±4.51ab	33.69±5.91ab	33.73±2.3ab	28.03±1.49b
63	Linalool	78-70-6	1100.59	52.12±4.01a	50.55±5.77a	50.73±6.02a	48.99±1.26a	52.59±4.75a
64	(-)-Rose oxide	3033-23-6	1110.00	12.1±0.45a	11.94±0.6a	11.58±0.62a	11.38±0.18a	11.83±0.23a
65	2-Methylisoborneol	2371-42-8	1198.00	21.99±0.42a	21.82±0.97a	23.35±0.76a	22.27±0.66a	23±1.36a
66	Carveol	99-48-9	1219.00	1.67±0.08a	0.61±0.08b	0.7±0.04b	0.65±0.01b	0.68±0.07b
67	Geraniol	106-24-1	1255.61	8.52±0.82a	8.5±0.16a	8.94±1.21a	8.42±0.12a	8.55±0.36a
68	Safranal	116-26-7	1205.72	17.86±0.5a	18.2±0.59a	18.13±0.21a	18.79±0.47a	18.6±1.44a
69	.alpha.-Terpineol	98-55-5	1195.55	45.92±1.32a	46.37±4.92a	49.39±2.29a	46.65±2.09a	50.69±6.18a
70	(-)-.β.-Bourbonene	5208-59-3	1384.00	0.43±0.03a	0.31±0.02b	0.25±0.02c	0.16±0.03d	0.2±0.01d
71	.alpha.-Ionone	127-41-3	1435.12	0.35±0.01a	0.39±0.06a	0.4±0.02a	0.35±0.05a	0.36±0.06a
72	(E)-alpha-bergamotene	13474-59-4	1435.00	0.47±0.04c	0.65±0.1bc	0.62±0.01bc	0.83±0.15b	1.06±0.17a
73	isocaryophyllene	118-65-0	1406.00	0.04±0.04c	0.11±0.01b	0.12±0b	0.15±0.02ab	0.18±0.04a
74	α-caryophyllene	6753-98-6	1466.78	0.11±0.07b	0.09±0.11b	0.23±0.01a	0.24±0.02a	0.25±0.04a
75	Cubenene	29837-12-5	1532.00	0.14±0.01a	0.15±0.01a	0.14±0.01a	0.06±0b	0.09±0.04b
76	β-sesquiphellandrene	20307-83-9	1524.00	0.91±0.49b	1.96±0.09a	1.21±0.69b	0.95±0.03b	0.72±0.19b
77	(-)-Myrtenol	515-00-4	1195.00	11.22±0.24a	11.17±1.3a	12.25±0.56a	11.62±0.53a	12.48±1.67a
78	5-methyl-2-(propan-2-yl)phenyl acetate	528-79-0	1355.00	0.13±0.01a	1.07±0.36a	0.76±0.07a	0.86±0.17a	1.14±0.2b

79	4,8,12-trimethyltrideca-1,3,7,11-tetraene	62235-06-7	1580.69	0.22±0c	0.31±0.08b	0.32±0.01b	0.34±0.03b	0.43±0.06a
80	Curzerenone	20493-56-5	1590.00	0.94±0.12c	1.6±0.27b	1.37±0.2b	1.36±0.06ab	1.81±0.19a
81	4-Methyl-2-(2-methyl-1-propenyl)tetrahydropyran	16409-43-1	1112.96	12.1±0.45a	11.94±0.6a	11.58±0.62a	11.38±0.18a	11.83±0.23a
82	Nerolidol	7212-44-4	1537.04	1.38±0.1a	1.27±0.65a	1.8±0.04a	0.71±0.02b	0.65±0.08b
83	terpinyl propionate	80-27-3	1432.00	4.26±0.14a	3.63±0.24a	3.18±0.05ab	1.87±1.48bc	1.07±1.41c
84	Ocimene	13877-91-3	1037.00	39.89±4.35a	35.51±4.51ab	33.69±5.91ab	33.73±2.3ab	28.03±1.49b
85	Iedane	28580-43-0	1373.00	0.45±0.02b	1.33±1.22b	2.89±0.1a	2.95±0.3a	2.78±0.38a
86	Panaxene	871660-95-6	1314.00	0.71±0.05a	0.71±0.04a	0.4±0.01b	0.37±0.03b	0.36±0.09b
Hydrocarbons								
87	1,2-Dimethylnaphthalene	573-98-8	1452.00	0.1±0.11ab	0.03±0b	0.1±0.13ab	0.23±0.02a	0.09±0.11ab
88	1,8-Dimethylnaphthalene	569-41-5	1472.00	0.03±0a	0.03±0a	0.07±0.09a	0.12±0.08a	0.06±0.07a
89	1,1,6-trimethyl-1,2-dihydro-Naphthalene	30364-38-6	1354.00	3.33±0.22a	3.08±0.23a	3.24±0.13a	3.29±0.14a	3.06±0.37a
90	2-Methoxy-4-vinylphenyl acetate	46316-15-8	1452.00	0.06±0b	0.06±0b	0.35±0a	0.33±0.01a	0.32±0.05a
91	n-Octadecane	593-45-3	1799.12	0.26±0.24a	0.28±0.25a	0.13±0.02a	0.26±0.21a	0.44±0.28a
92	5-methyldecane	13151-35-4	1057.00	4.68±0.8a	2.99±1.31ab	2.18±0.65b	2.16±0.6b	2.99±1.27ab
93	2,4-Decadienal	2363-88-4	1319.21	0.06±0b	1.34±0.9a	0.49±0.04b	0.53±0.04b	0.56±0.02b
Ketone								
94	2-Dodecanone	6175-49-1	1396.00	1.32±0.13ab	1.08±0.74ab	1.58±0.22a	1.56±0.13ab	0.63±0.71b

95	(E)-alpha-damascone	24720-09-0	1395.00	0.02±0c	0.02±0c	0.02±0c	0.12±0.01b	0.19±0.01a
96	piperitenone oxide	35178-55-3	1368.00	0.3±0.03c	0.41±0.06bc	0.4±0.04bc	0.47±0.05b	0.59±0.1a
97	(E)-filbertone	102322-83-8	972.00	0.03±0a	0.03±0a	0.23±0.18a	0.14±0.09a	0.18±0.13a
98	3-Octen-2-one	1669-44-9	1040.00	0.24±0.04a	0.28±0.03a	0.3±0.08a	0.31±0.04a	0.31±0.08a
	3,5-Octadien-2-one	38284-27-4	1091.00	25.26±0.95a	23.74±2.67a	24.3±1.36a	22.63±1.98a	25.04±0.36a
99	2-Undecanone	112-12-9	1294.65	0.02±0d	0.11±0.01c	0.29±0.02b	0.37±0.03a	0.38±0.03a
100	Phenylacetone	103-79-7	1124.00	1.48±0.5d	2.35±0.52cd	3.11±0.21bc	3.92±0.27ab	4.74±0.94a
101	<u>Ethyl cyclopropyl ketone</u>	6704-19-4	756.00	0.06±0.05ab	0.1±0.02a	0.05±0.04ab	0.04±0b	0.02±0.02b
102	3,5-Octadien-2-one	30086-02-3	1073.00	25.26±0.95a	23.74±2.67a	24.3±1.36a	22.63±1.98a	25.04±0.36a
103	Dihydro-β-ionone	17283-81-7	1433.00	0.04±0.03c	0.08±0.01b	0.08±0.01b	0.1±0.02b	0.15±0.02a
104	Gallacetophenone	528-21-2	1453.00	0.02±0c	0.04±0b	0.05±0.01a	0.05±0a	0.06±0.01a
105	2-Methyl-1-penten-3-one	25044-01-3	748.00	0.06±0.05ab	0.1±0.02a	0.05±0.04ab	0.04±0b	0.02±0.02b
106	<u>2-Tridecanone</u>	593-08-8	1495.82	0.15±0.01c	0.09±0.07c	0.48±0.02b	0.72±0.06a	0.68±0.05a
107	<u>2-Pentadecanone</u>	2345-28-0	1698.33	0.07±0.01a	0.08±0b	0.16±0.01c	0.25±0.01d	0.31±0.05d
108	2-Dodecanone	6175-49-1	1396.00	1.32±0.13ab	1.08±0.74ab	1.58±0.22a	1.56±0.13ab	0.63±0.71b
Heterocyclic compound								
109	2-(12-Tridecynyl)furan	24708-33-6	1827.00	0.01±0.01b	0.04±0.02a	0.03±0.01ab	0.03±0.01ab	0.03±0.01ab
110	2-Methyl-3-(methylthio)pyrazine	2882-20-4	1184.00	1.13±0.02a	0.95±0.12b	1.14±0.08a	1.09±0.09ab	1.13±0.09a
111	2-Ethoxy-3-methylpyrazine	32737-14-7	1065.00	1.1±0.05ab	1.12±0.12a	1.1±0.08ab	1.13±0.06a	0.97±0.03b
112	methylfurfurylthiol,5-methyl-2-furfurylthiol	59303-05-8	995.00	3.76±0.13a	3.77±0.71a	3.95±0.68a	4.48±0.64a	4.63±0.63a

113	2-Thiophenemethanethiol	6258-63-5	1105.00	12.19±0.54a	12.94±0.82a	11.91±1.26a	12.09±0.93a	13.07±0.29a
114	3,5-Dimethyl-2-ethylpyrazine	13925-07-0	1084.00	0.2±0.02a	0.19±0.04a	0.19±0.07a	0.21±0.02a	0.17±0.05a
115	2-Acetylthiophene	88-15-3	1092.00	6.63±0.65a	6.49±0.97a	6.3±0.81a	6.31±0.1a	6.81±0.68a
116	2-Pentylpyridine	2294-76-0	1202.00	0.68±0.04a	0.69±0.07a	0.73±0.05a	0.69±0.02a	0.73±0.09a
117	3-Hydroxy-4-methyl-5-ethyl-2(5H)furanone	698-10-2	1195.00	15.84±1.72a	15.84±2.09a	15.9±2.13a	17.23±0.28a	17.13±0.18a
118	Apricolin	104-61-0	1368.03	1.76±1.45a	1.51±0.91a	1.96±1.3a	0.9±0.14a	2.04±1.39a
119	5-ethylfuran-2(5H)-one	2407-43-4	966.00	21.03±1.2ab	21.54±1.19a	20.79±0.89ab	22.02±0.22a	19.09±2.02b
120	Myosmine	532-12-7	1431.01	0.04±0a	0.02±0.01a	0.03±0a	0.11±0.07a	0.12±0.09a
121	Anabasine	494-52-0	1514.00	0.08±0ab	0.1±0.03ab	0.05±0.07b	0.09±0.01ab	0.13±0.01a
122	GrAmine	87-52-5	1620.00	0.06±0.01c	0.08±0.01c	0.1±0b	0.13±0.01a	0.13±0.02a
123	Isonicotinonitrile	100-48-1	978.00	1.11±0.18a	1.05±0.07a	0.52±0.13b	0.23±0.02c	0.16±0.03c
124	5-Hydroxyoctanoic acid lactone	698-76-0	1288.00	1.11±0.22a	1.27±0.17a	1.2±0.24a	1.14±0.22a	1.29±0.51a
125	4-Aminopyrazolo[3,4-d]pyrimidine	2380-63-4	1595.00	0.25±0.12b	0.32±0.01b	0.56±0.04a	0.4±0.17ab	0.37±0.04ab
126	2-thiophen-3-ylpyridine	21298-55-5	1362.00	0.14±0.01c	0.22±0.05b	0.22±0.01b	0.28±0.01ab	0.34±0.07a
127	1-(1H-Imidazol-4-yl)-1-pentanone	69393-15-3	1371.00	0.07±0.01c	0.06±0.05c	0.12±0.01b	0.17±0.02ab	0.19±0.03a
128	(4S,5R)-5-hexyl-4-methyloxolan-2-one	147254-33-9	1444.00	0.29±0.03a	0.16±0.18a	0.35±0.04a	0.34±0.02a	0.15±0.16a
129	1-Ethyl-1,2-dihydro-3H-indazol-3-one	54385-62-5	1422.00	0.2±0.02a	0.12±0.02b	0.09±0c	0.07±0.02c	0.06±0d
130	3-n-propyl-2-pyrazolin-5-one	29211-70-9	1071.00	0.92±0.45a	0.46±0.08a	0.74±0.58a	0.91±0.23a	0.77±0.16a

131	Furylacrolein	623-30-3	1111.00	0.38±0.01d	0.51±0.04c	0.61±0.06bc	0.71±0.02b	0.87±0.11a
132	3-Furaldehyde	498-60-2	831.00	0.38±0.06a	0.3±0.07a	0.01±0b	0.09±0.01b	0.09±0.07b
Ester								
133	Dihydrothymine	696-04-8	1523.27	0.06±0.01b	0.07±0a	0.08±0a	0.08±0.01a	0.07±0.01a
134	Gramine	87-52-5	1612	0.06±0.01c	0.08±0.01c	0.1±0b	0.13±0.01a	0.13±0.02a
135	3-cyanopyrrole	7126-38-7	1118	3.83±0.25a	3.28±0.25ab	3±0.47b	2.93±0.27b	2.87±0.61b
136	1,5-dihydro-pyrrolo [3,2-a]pyri	65996-50-1	756	0.2±0.02a	0.17±0a	0.17±0.02a	0.19±0.02a	0.18±0.03a
137	2-Butyl-5-propylthiazole	52414-86-5	1022	0.58±0.02b	0.68±0.1ab	0.73±0.04ab	0.8±0.1a	0.83±0.14a
138	Nicotine	54-11-5	1233	0.07±0.01b	0.1±0.02a	0.07±0.01b	0.06±0.01b	0.07±0.01b
139	2-Acetylpyrroline	60026-20-2	1402	0.4±0.02a	0.46±0.04a	0.46±0.08a	0.44±0.03a	0.44±0.06a
140	1H-Pyrrole-2-carbonitrile	9513-94-4	869	3.83±0.25a	3.28±0.25a	3±0.47b	2.93±0.27b	2.87±0.61b
141	1,1-Dimethylethyl (3S)-3[(phenylmethyl)amino]-1.pyrrolidinecarboxylate	134881-46-2	1447	3.63±0.07c	4.31±0.28bc	5.16±0.27ab	4.11±0.4c	5.36±0.97a
142	1,5-Diazabicyclo[3.1.0]hexane	13090-31-8	1395	2.3±0.12a	1.77±0.36b	1.37±0.05c	1.47±0.04bc	1.29±0.16c
143	(+)-JQ1 carboxylic acid	20295-23-2	1366	0.06±0.01a	0.08±0.01b	0.11±0.01a	0.11±0a	0.11±0.02c
144	2- (3-Thienyl)pyridine	21298-55-5	1392	0.14±0.01c	0.22±0.05b	0.22±0.01b	0.28±0.01ab	0.34±0.07a
145	1-Ethyl-1,2-dihydro-3H-indazol-3-one(1-Ethyl-1,2-dihydro-3H-indazol-3-one)	54385-62-5	1209	0.2±0.02a	0.12±0.02b	0.09±0c	0.07±0.02cd	0.06±0d
146	1H-Benzimidazole,2-methoxy	22128-99-0	1440	0.12±0.02b	0.17±0.03a	0.17±0.01a	0.14±0ab	0.17±0.04a
147	Pyrimidine, 4-(2-	98489-37-3	1079	15.36±0.35a	14.56±1.91ab	15.97±0.53a	13.51±0.43b	11.34±0.54c

	methylpropyl)							
148	1-Butyl-3,4-dihydroisoquinoline	33351-43-8	1479	0.44±0.01c	0.64±0.12b	0.62±0.03b	0.69±0.02ab	0.81±0.14a
149	Furylacrolein	623-30-3	1088	0.38±0.01d	0.51±0.04c	0.61±0.06bc	0.71±0.02b	0.87±0.11a
150	7 (1H)-Pteridinone	2432-27-1	1269	0.46±0c	0.78±0.06ab	0.66±0.03b	0.64±0.11b	0.87±0.14a
151	3-methyl-2(3H)-Benzofuranone	32267-71-3	1577	0.14±0.02a	0.13±0.01ab	0.11±0.01bc	0.1±0.01c	0.09±0.01c
152	2-Acetyl-3-Methylbenzo [b]Thiophene	18781-31-2	1168	3.44±0.44a	5.21±0.61ab	4.55±0.26b	4.85±0.36b	6.06±0.59c
153	(3-hydroxy-2-furyl)ethanone	3420-59-5	1092	0.1±0.01a	0.1±0.01a	0.1±0.01a	0.07±0b	0.08±0.01b
154	3-Phenylpropionic acid methyl ester	103-25-3	1279	0.21±0.01ab	0.23±0.04a	0.16±0.01bc	0.18±0.01bc	0.18±0.01c
155	Phenethyl isobutyrate	103-48-0	1396	0.07±0b	0.09±0.01b	0.09±0b	0.09±0.01b	0.12±0.02a
156	Cinnamyl isobutyrate	103-59-3	1586	0.62±0.01c	1±0.18b	0.88±0.03b	0.85±0.15b	1.29±0.13a
157	Bicyclo	29135-27-1	1279	1.65±0.08a	1.35±0.08b	1.38±0.08b	1.17±0.11b	1.26±0.18b
158	neryl isovalerate	3915-83-1	1582	0.07±0d	0.12±0b	0.1±0c	0.12±0b	0.15±0.02a
159	Benzyl acetate	140-11-4	1170.64	0.13±0.02e	0.18±0.01d	0.23±0.01c	0.36±0.03b	0.47±0.02a
160	Hexyl 2-methylbutyrate	10032-15-2	1261	0.5±0.03a	0.31±0.03b	0.26±0.02b	0.11±0.1c	0.04±0.01c
161	Phenethyl acetate	103-45-7	1252	0.09±0.02c	0.1±0.01c	0.16±0.01b	0.17±0b	0.49±0.05a
162	bornyl formate	7492-41-3	1240	0.2±0.01b	1.43±0.2a	1.41±0.26a	1.29±0.2a	1.49±0.31a
163	Methyl 2-aminobenzoate	134-20-3	1306	1.81±0.14a	1.54±0.09b	1.37±0.1bc	1.3±0.1c	1.17±0.13c
164	n-Butyl benzoate	136-60-7	1295.30	0.19±0.01a	0.17±0.01ab	0.16±0.01bc	0.13±0.01c	0.13±0.02c
165	Ethyl 3-phenylpropanoate	2021-28-5	1367	4.59±0.68c	6±0.32b	6.69±0.59b	7±0.28b	8.86±0.95a
166	α-Terpinyl acetate	80-26-2	1353	0.2±0.01d	0.27±0.05c	0.27±0.02c	0.34±0.01b	0.42±0.05a
167	Nerylacetate	141-12-8	1366	1.26±0.11a	0.83±0.31b	0.76±0.15b	0.67±0.18b	0.62±0.11b

168	linalyl butyrate	78-36-4	1276.46	0.77±0.05a	0.62±0.03b	0.51±0.03bc	0.54±0.04bc	0.44±0.12c
169	citronellyl valerate	7540-53-6	1576	0.34±0.01b	0.51±0.11a	0.47±0.01a	0.53±0.02a	0.57±0.08a
170	(-)-methyl jasmonate	1211-29-6	1625	0.4±0.17b	0.81±0.15a	0.77±0.03a	0.83±0.05a	0.89±0.15a
171	Ethyl tetradecanoate	124-06-1	1478	0.14±0b	0.16±0.01ab	0.17±0.01ab	0.19±0.01a	0.19±0.04a
172	δ-Dodecanolactone	713-95-1	1793	0.63±0.13c	0.83±0.17bc	0.8±0.1bc	1.17±0.23ab	1.49±0.5a
173	L(+)-Diethyl L-tartrate	87-91-2	2024	0.39±0.03c	0.51±0.03bc	0.56±0.04b	0.71±0.03a	0.8±0.16a
174	Cyclohexyl acetate	622-45-7	1013.99	28.66±3.14a	20.72±0.83c	22.27±0.8bc	23.83±0.72b	19.77±0.78c
175	(±)-alpha-terpinyl acetate	10235-63-9	1462	0.2±0.01d	0.27±0.05c	0.27±0.02c	0.34±0.01b	0.42±0.05a
176	3-phenylpropyl acetate	122-72-5	1367	0.1±0.01c	0.12±0.02b	0.13±0.01b	0.14±0.02b	0.17±0.02a
177	Remonol Euresol	102-29-4	1381	0.2±0.01a	0.18±0.01b	0.16±0bc	0.17±0b	0.14±0.01c
178	Cinnamylacetate	21040-45-9	1446	0.07±0.01a	0.08±0.01a	0.08±0a	0.07±0.01a	0.08±0a
179	(E)-2-Hexenyl benzoate	76841-70-8	992.96	1.72±0.03b	2.78±0.5a	2.53±0.12a	2.72±0.23a	3.21±0.67a
180	Methyl undecanoate	1731-86-8	1308	0.03±0c	0.05±0b	0.07±0a	0.05±0b	0.05±0.01b
181	Ethyl (E)-4-decenoate	76649-16-6	1432	0.04±0.01d	0.06±0.01c	0.07±0.01bc	0.08±0b	0.09±0.01a
182	3,5,5-Trimethylcyclohexyl methacrylate	7779-31-9	1516	0.3±0.03c	0.42±0.03b	0.4±0.02b	0.46±0.04b	0.57±0.09a
183	Carbonic acid, phenyl propyl ester	13183-16-9	1581.00	0.55±0.05a	0.48±0.04b	0.45±0.02bc	0.4±0.04c	0.38±0.04c
184	Hexanoic acid	108058-81-7	1171	0.33±0.02a	0.27±0.02b	0.25±0.01b	0.26±0b	0.25±0.04b
185	Methyl 3,4-dimethylbenzoate	38404-42-1	1355.00	5.17±0.78	7.55±1.74	7.59±0.55	8.62±1.72	11.4±1.91

Table S3. Relative Odor Activity Values (rOAV ≥ 1) of Volatile Compounds during Fermentation of peach and apricot mixed juice

Compounds	Threshold(μg/L)	0h	8	12	16	20	Description
3-Hydroxy-4-methyl-5-ethyl-2(5H)furanone	0.002	7920.44	7917.51	7952.01	8615.48	8564.13	sweet, fruity, caramel
(Z)-6-Nonenal	0.140	551.83	546.31	534.70	526.65	550.72	green, cucumber, melon
(Z,Z)-3,6-Nonadienal	0.050	452.23	482.26	434.38	432.95	430.00	fatty, soapy, cucumber
2-Thiophenemethanethiol	0.040	304.76	323.39	297.70	302.20	326.68	roasted, coffee, fishy
2-phenylethyl3-methylbutirate	0.010	184.86	197.26	185.25	209.23	211.29	floral, fruity, sweet
γ-Decalactone	1.100	93.34	94.38	108.05	104.22	96.90	fresh, oily, waxy
methylfurfurylthiol,5-methyl-2-furfurylthiol	0.050	75.23	75.42	79.05	89.54	92.68	sulfury, roasted, coffee
(-)-Rose oxide	0.200	60.48	59.70	57.91	56.89	59.14	rose, cortex, green,
4-Methyl-2-(2-methyl-1-propenyl)tetrahydropyran	0.200	60.48	59.70	57.91	56.89	59.14	sweet, floral, aromatic
3,5-Octadien-2-one	0.500	50.52	47.47	48.59	45.25	50.08	fruity, green, grassy
3-Mercapto-3-methylbutyl Formate	0.002	77.07	56.68	73.68	46.17	31.40	sulfury, catty, caramel
2-Methylisoborneol	0.480	45.81	45.46	48.66	46.40	47.92	earthy, musty
Methyl benzoate	0.520	32.07	33.88	34.34	31.49	34.77	phenol, wintergreen
(2E,4E)-Deca-2,4-dienal	0.070	0.89	19.10	6.99	7.61	8.01	dusty, waxy, oily
Dodecanenitrile	0.090	23.46	24.94	24.25	26.68	26.93	citrus, orange, peel
Eugenol	2.500	18.79	24.87	23.34	27.83	34.60	floral, clove
Cyclohexyl acetate	1.600	17.91	16.28	14.75	14.89	12.36	fruity, sweet, musty
Nonanal	1.000	13.45	13.37	9.88	10.39	11.40	aldehyde, citrus
3-Octen-2-one	0.030	8.12	9.33	10.00	10.39	10.31	earthy, spicy, herbal

Benzyl isothiocyanate	0.700	7.15	10.10	10.02	12.04	14.74	mild, watercress, dusty
Linalool	6.000	8.69	8.42	8.46	8.17	8.77	floral, green
Dimethyl trisulfide	0.008	15.04	10.94	8.09	4.23	8.28	sulfury, cooked onion
3-Ethylphenol	0.850	10.70	10.88	26.31	30.78	27.42	musty
2-Acetylthiophene	1.000	6.63	6.49	6.30	6.31	6.81	sulfury, nutty, hazelnut
Safranal	3.000	5.95	6.07	6.04	6.26	6.20	fresh, herbal, phenol
3,5-Dimethyl-2-ethylpyrazine	0.040	4.97	4.65	4.85	5.23	4.29	burnt, almond, roasted
3-Mercaptohexyl acetate	0.020	4.55	4.95	4.99	4.83	4.72	sulfury, grapefruit, fruity
Ethyl methyl disulfide	0.062	3.67	3.64	4.16	3.51	4.39	sulfury, truffle
(2E,4Z)-2,4-Decadienal	0.070	10.46	3.08	1.02	1.05	1.09	fried, fatty, geranium
(E)-4-Nonenal	2.200	2.28	2.28	2.30	2.24	2.40	fruity
3-Mercaptohexanol	0.060	1.89	2.39	2.70	2.84	3.83	sulfury, fruity, tropical
5-ethylfuran-2(5H)-one	9.700	2.17	2.22	2.14	2.27	1.97	spice
(-)-Myrtenol	7.000	1.60	1.60	1.75	1.66	1.78	woody, minty
2-Ethoxy-3-methylpyrazine	0.800	1.38	1.40	1.38	1.42	1.21	hazelnut, roasted, almond
3-Methylthiopropanol	36.000	1.49	1.41	0.69	0.27	0.22	cabbage, cooked potato
Phenylacetaldehyde	6.300	1.40	1.12	0.84	0.85	0.81	floral, honey, rose
1,1,6-trimethyl-1,2-dihydro-Naphthalene	2.500	1.33	1.23	1.30	1.32	1.22	Licorice
Geraniol	6.600	1.29	1.29	1.35	1.28	1.30	sweet, floral, fruity
.beta.-Myrcene	15.000	0.95	1.07	1.14	1.16	1.29	musty, balsamic, spice
p-Anisaldehyde	0.200	1.31	1.32	1.13	1.04	1.11	sweet, powdery, mimosa
2-Pentylpyridine	0.600	1.14	1.14	1.22	1.15	1.22	fatty, tallow, green
6-nonenal	0.022	1.09	1.09	7.21	6.37	8.20	-
Methyl laurate	3.500	1.06	1.12	1.39	1.94	1.28	waxy, soapy, creamy

Ocimene mixture of isomers	34.000	1.17	1.04	0.99	0.99	0.82	warm, floral, herbal
Ocimene	34.000	1.17	1.04	0.99	0.99	0.82	apple, pear, fruity
D-Limonene	34.000	1.15	1.04	0.95	0.96	0.79	citrus
1-Nonanol	5.300	0.71	0.80	1.04	1.05	1.19	fresh, clean, fatty
2-Methyl-3-(methylthio)pyrazine	1.000	1.13	0.95	1.14	1.09	1.13	roasted meat, nutty
(E)-filbertone	0.050	0.69	0.69	4.68	2.76	3.61	hazelnut, nutty
γ-Octanoic lactone	17.900	0.37	0.39	0.38	0.35	0.37	sweet, coconut, waxy
Nonanoic acid	1.600	0.30	0.31	0.53	0.67	1.00	waxy, dirty, cheese

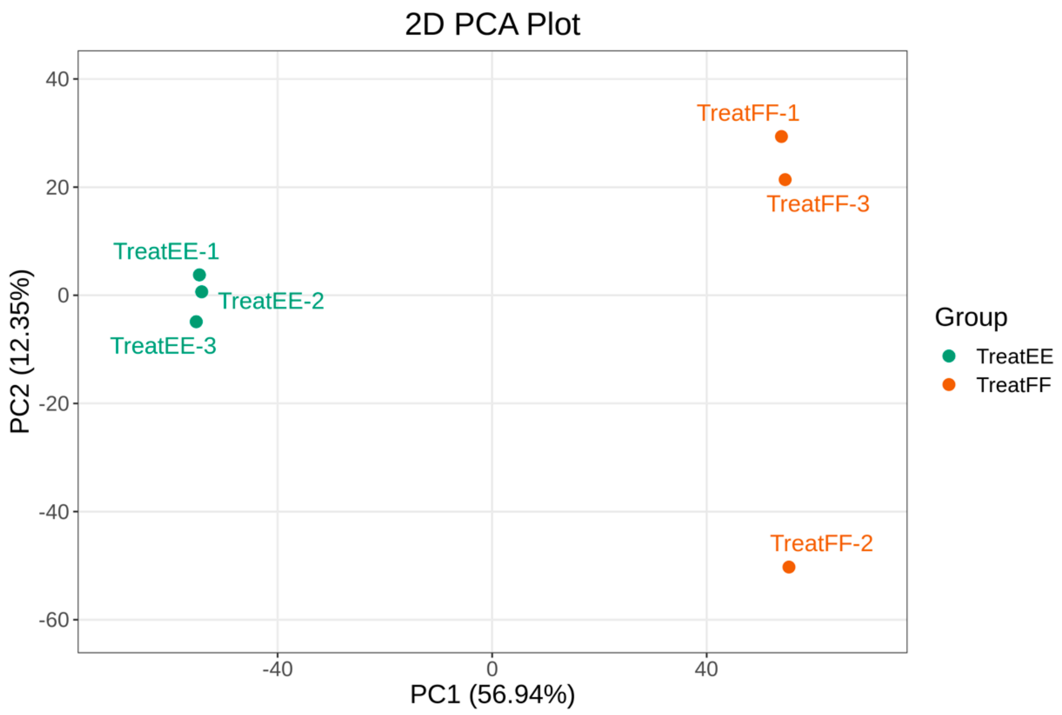

Figure S1. partial least squares analysis of non-volatile metabolite variations during fermentation
